# Naturalistic stimulation changes the dynamic response of action potential encoding in a mechanoreceptor

**DOI:** 10.3389/fphys.2015.00303

**Published:** 2015-10-30

**Authors:** Keram Pfeiffer, Andrew S. French

**Affiliations:** Department of Physiology and Biophysics, Dalhousie UniversityHalifax, NS, Canada

**Keywords:** mechanotransduction, information transmission, neuronal coding, naturalistic, spider

## Abstract

Naturalistic signals were created from vibrations made by locusts walking on a *Sansevieria* plant. Both naturalistic and Gaussian noise signals were used to mechanically stimulate VS-3 slit-sense mechanoreceptor neurons of the spider, *Cupiennius salei*, with stimulus amplitudes adjusted to give similar firing rates for either stimulus. Intracellular microelectrodes recorded action potentials, receptor potential, and receptor current, using current clamp and voltage clamp. Frequency response analysis showed that naturalistic stimulation contained relatively more power at low frequencies, and caused increased neuronal sensitivity to higher frequencies. In contrast, varying the amplitude of Gaussian stimulation did not change neuronal dynamics. Naturalistic stimulation contained less entropy than Gaussian, but signal entropy was higher than stimulus in the resultant receptor current, indicating addition of uncorrelated noise during transduction. The presence of added noise was supported by measuring linear information capacity in the receptor current. Total entropy and information capacity in action potentials produced by either stimulus were much lower than in earlier stages, and limited to the maximum entropy of binary signals. We conclude that the dynamics of action potential encoding in VS-3 neurons are sensitive to the form of stimulation, but entropy and information capacity of action potentials are limited by firing rate.

## Introduction

Gaussian random noise is widely used as a stimulus signal for linear and nonlinear characterization of biomedical systems, including neurons and neuronal coding (French and Holden, 1971; Marmarelis, 1994; French and Marmarelis, 1999; DiCaprio et al., 2007). Important reasons for its use are the efficiency of measuring a wide range of frequencies simultaneously, and its similarity to the unpredictable range of signals that many biological systems encounter in the natural world. However, many natural stimuli are not completely random but instead contain limited frequency bands and predictable components. This is not important for characterizing linear systems, whose dynamics are independent of input, but may be crucial for nonlinear systems, such as sensory neurons. Therefore, it is important to consider how neural systems respond to naturalistic signals, and whether neuronal coding has evolved to maximize the information available in the natural signals that each neuron receives and processes.

Responses to naturalistic inputs, which feature all or parts of the properties of natural signals, have been studied in several sensory modalities. Frog low frequency sensitive auditory fibers gave higher signal-to-noise values and more efficient entropy values when Gaussian noise was limited to a similar frequency envelope as natural calls (Rieke et al., 1995) and synaptic decoding of natural birdsong was dependent on its time-dependent structure (Mehaffey and Doupe, 2015). Naturalistic sound stimuli have also been used to explore insect central nervous system organization and function (Dupuy et al., 2012). Fly second-order visual cells adjusted their sensitivity to wide dynamic intensity naturalistic signals by nonlinear sensitivity compression (van Hateren, 1997). Variations in amplitude and temporal sensitivities caused by naturalistic inputs were further studied in fly photoreceptors (Juusola and de Polavieja, 2003), with identification of nonlinear contributions to dynamic control by voltage-activated ion channels (Niven et al., 2004), as well as feed-forward and feedback synaptic connections between different cell layers in the retina (Nikolaev et al., 2009; Zheng et al., 2009). Naturalistic visual flow patterns have been used to test the effects of octopaminergic neural modulation in insects (Rien et al., 2013).

A general question of these studies is how sensory systems have evolved to extract information optimally from their natural signals, although there must also be a wide spectrum of functional adaptations, from systems highly tuned to specific predator or prey signals to those that cope with much wider ranges of inputs. Quantifying these adaptations is challenging because the relative importance of inputs and neuronal functions are often poorly understood. Nevertheless, the nonlinearity of most sensory systems makes it important to consider their responses to naturalistic signals.

Spider lives are often dominated by mechanical inputs and events (Barth, 2002). The wandering spider, *Cupiennius salei*, waits on the leaves of plants for substrate vibrations from prey insects, it also uses direct and substrate vibration for communication of mating signals (Barth, 2002; Molina et al., 2009). We created naturalistic mechanical signals by measuring vibrations produced by locusts walking on a *Sansevieria* plant. Using intracellular current clamp and voltage clamp recordings, we compared the responses of VS-3 slit sensilla neurons to naturalistic and Gaussian noise signals at the receptor current, receptor potential and action potential levels. While naturalistic stimuli have been used before to measure responses in the metatarsal lyriform organ of this spider (Molina et al., 2009), our study is the first to analyze this kind of experiment using a systems analysis approach and compare frequency responses between naturalistic and random stimuli.

Sensitivity was lower for naturalistic signals at each stage of sensory processing, but the strongest effect was an increase in relative sensitivity to high frequencies in the action potential signals. Comparing the signal entropies and information capacities at each stage, and with both types of stimulus, indicated that uncorrelated noise is added to the receptor current. Finally, we compared adapted and unadapted neurons receiving identical levels of Gaussian stimulation, but found that neither firing rate nor adaptation state changed the frequency response.

## Materials and methods

### Animals and preparation

**Figure 1:**
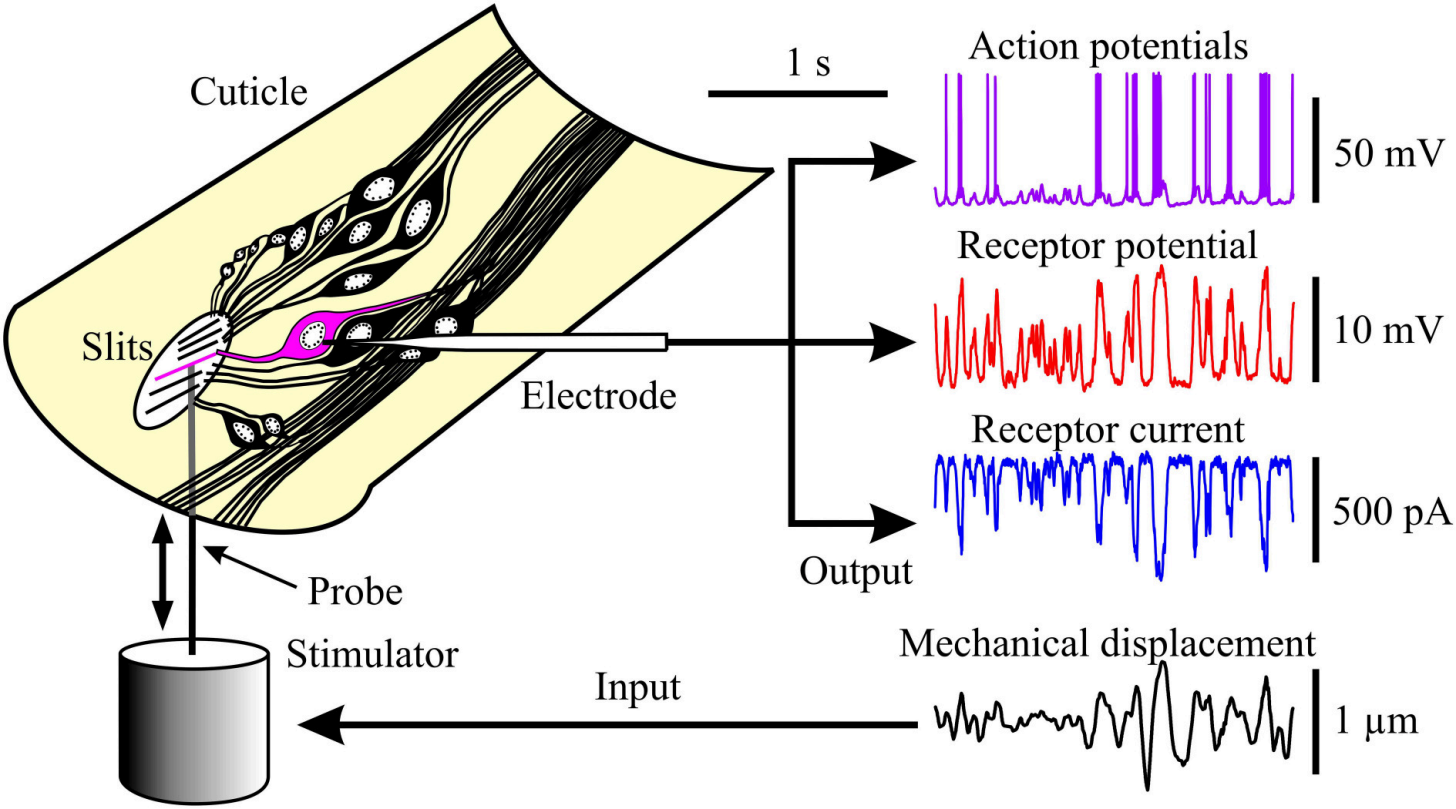
**Experimental arrangement for observing the three stages of mechanoreception in spider VS-3 neurons**. **Left:** neurons of a VS-3 slit-sense organ exposed in a piece of patella cuticle. Mechanical stimulation with a naturalistic sequence fed via a piezo-electric actuator (fourth trace on the right) caused electrical activity in a neuron that was detected by an intracellular microelectrode. Other traces on the **Right:** action potentials under current clamp (first trace), receptor potential under current clamp after action potentials were blocked with tetrodotoxin (TTX; second trace), and receptor current under voltage clamp with TTX (third trace).

Experiments were performed on VS-3 patella slit sense organs obtained from adult female tropical wandering spiders (*Cupiennius salei*). Spiders were reared in our laboratory colony at 22 ± 2°C under a 13:11-h light-dark cycle. Legs were autotomized according to a protocol approved by the Dalhousie University Committee on Laboratory Animals. A piece of patella cuticle containing the entire VS-3 slit sense organ was dissected from the leg and waxed to a petri dish (Figure 1). The petri dish had a small hole beneath the cuticle to allow access from below (stimulation) and above (recording). The preparation was continually superfused with spider saline (pH 7.8) containing (in mM) 223 NaCl, 6.8 KCl, 8 CaCl2, 5.1 MgCl2, 10 HEPES, and 17 glucose.

### Electrophysiology

Neurons were visualized using a fixed stage upright microscope (Zeiss Axioskop 2 FS plus. Oberkochen, Germany) with a 40X water immersion lens (Zeiss Achroplan), mounted on a gas-driven vibration isolation table (Technical Manufacturing, Peabody, PA). Sharp microelectrodes were pulled from omega-shaped borosilicate glass (outer diameter: 1 mm, inner diameter 0.5 mm, Hilgenberg, Malsfeld, Germany) using a laser heated horizontal puller (P-2000, Sutter Instrument, Novato, CA). Electrodes were filled with 2.5 M KCl and had resistances of 35–75 MΩ in solution. Neurons were impaled by advancing the electrodes with a Patch Star micromanipulator (Scientifica, Uckfield, East Sussex, UK) and by overcompensation of the amplifier (SEC-10LX, npi, Tamm, Germany). Action potentials and receptor potentials were recorded in discontinuous single-electrode current clamp mode. Receptor current was measured in discontinuous single-electrode voltage clamp (SEVC) mode. Receptor potential and current recordings were made during superfusion with 1 μM Tetrodotoxin (Ascent Scientific, Cambridge UK). In SEVC mode, the neurons were clamped at their resting potential (−70 to −80 mV). Switching frequency of the amplifier was set between 18 and 20 kHz at a duty cycle of 1/4 (current passing/voltage recording). Voltage and current were low-pass filtered at 33.3 and 3.3 kHz respectively. Conditions for successful current- and voltage-clamp recording using the switching method have been described previously (Weckström et al., 1992) as well as their application to *Cupiennius* VS-3 neurons (Juusola et al., 1994). Data acquisition and stimulation were controlled by a personal computer using custom-written software. Data was sampled at 16 bits and stimulus control was at 12 bits, both at 10 kHz via a data acquisition board (National instruments, Austin, TX).

### Stimulation

Mechanical stimulation was performed by pushing a small glass probe against the leg cuticle in the region of the slits using a P-841.10 piezoelectric stimulator driven by an E-505.00 LVPZT amplifier (Physik Instrumente, Auburn, MA). We employed three different stimulus protocols. In all cases, the command input was effectively low pass filtered with a corner frequency of ~70 Hz and maximum mechanical signal frequency of ~300 Hz by the electromechanical properties of the stimulator and its servo loop controller. In the basic (adapting) stimulation, the stimulator was driven with Gaussian white noise, created from a 33-bit maximum binary sequence algorithm with a time resolution of 0.1 ms. Stimulation lasted at least 300 s. To measure the response properties of virtually unadapted VS-3 neurons to the same stimulus, we stimulated for 4 s every 29 s (i.e., 25 s pause between start and end of successive repetitions). This was repeated 25 times to yield 100 s of neuronal response.

To obtain a naturalistic stimulus signal, we measured vibrations elicited by fifth instar larvae of the desert locust (*Schistocerca gregaria*) walking up the leaves of a *Sansevieria trifasciata* plant, using laser Doppler vibrometry. While *Schistocerca gregaria* does not occur in the natural habitat of *Cupiennius* its habitus and locomotion are very similar to those of other acridid species that are potential prey insects for wandering spiders. *Sansevieria* plants have long, thick and sturdy leaves and have been described to be amongst typical habitat plants for *Cupiennius* (Barth, 2002). Individual insects were placed at the base of the plant. Showing negative gravitactic response, locusts usually slowly walked up the plant. Vibrations caused by the locust's motion were measured as the linear displacement of the base of the plant over time using a PSV-400 scanning laser Doppler vibrometer (Polytec, Waldbronn, Germany). Signals were sampled at 30 kHz using a CED 1401 micro and Spike 2 (both Cambridge Electronic Design, Cambridge, UK). To obtain a stimulus of 150 s duration, signals from eight runs were concatenated, low-pass filtered at 5 kHz and then resampled at 10 kHz. The resulting trace was then used to drive the piezoelectric stimulation system in all recordings. For each recording the stimulus was presented twice, yielding a total recording time of 300 s each.

### Frequency responses

Frequency response function estimation in VS-3 neurons has been described previously in detail (French et al., 2001; Torkkeli et al., 2012). Mechanical stimulus, receptor current and receptor potential signals were all resampled at 1 kHz to give a frequency range of 0–500 Hz. Action potentials were initially sampled at 10 kHz, then times of occurrence were treated as Dirac delta functions, and digitally filtered with a sin(x)/x function and re-sampled at 1 kHz to band-limit the frequency range to 0–500 Hz (French and Holden, 1971), giving a maximum frequency above that of the mechanical stimulator (see above) and above the maximum possible firing rate because action potentials were ~4 ms duration.

Input and output signals were transferred to the frequency domain using the fast Fourier transform (Cooley and Tukey, 1965) in segments of 1024 input–output data pairs. Frequency response functions were calculated from cross-spectral and auto-spectral estimates (Bendat and Piersol, 1980) and plotted as Bode plots (phase and log gain amplitude vs. log frequency) by averaging exponentially increasing numbers of spectral estimates with frequency to give approximately equally spaced data points on a logarithmically scaled x-axis.

Frequency response functions between displacement and action potentials were fitted by the power-law relationship (French et al., 2001):

(1) $$\begin{array}{r} G\left(\omega \right)=A\omega^{k}, \end{array}$$

(2) P(ω)=k90°,

where ω is frequency, *G*(ω) is the real amplitude of the complex frequency response, *P*(ω) is the phase lag, *A* represents overall sensitivity (gain at 1 Hz), and *k* is a fractional exponent of frequency. Fitting was performed on the frequency response function in complex exponential format.

Responses between displacement and receptor potential or receptor current were fitted by an equation for a simple closed cable (Jack et al., 1983):

(3) $$\begin{array}{r} G\left(\text{j}\omega \right)=\alpha ∕\text{cosh}\left(\left(x∕\lambda \right)\sqrt{}\left(j\omega \tau +1\right)\right), \end{array}$$

where *G*(jω) is the complex frequency response, $\text{j}=\sqrt{-}1$, α is the scale of transduction from mechanical displacement to electric current or potential, *x* is distance along the cable, λ is cable space constant and τ is cable time constant. To simplify fitting, τ was fixed at 6.25 ms based on earlier cable measurements (Gingl and French, 2003) and *x*/λ was treated as a single combined parameter.

The coherence function, γ^2^ (ω) (Bendat and Piersol, 1980) between input and output signals was calculated for each frequency response and used to estimate the linear information capacity, *R* (Shannon and Weaver, 1949):

(4) $$R=\int log_{2}[1/(1-\gamma^{2}(\omega ))]d\omega .$$

### Entropy

Entropy in the stimulus and the recorded traces was estimated by the context-free data compression approach described previously (French and Pfeiffer, 2011; Pfeiffer et al., 2012). Action potential times of occurrence were rewritten as a sequence of two binary symbols representing either the occurrence of a spike (1) or no spike (0) with a time resolution of 1 ms. Receptor current and receptor potentials were resampled and represented by 1024 symbols, i.e., each point in time was given a value between 0 and 1023 (10 bit values). The resulting traces were then searched for combinations of symbols that occurred with the highest frequency. These symbols were replaced by new symbols. The process was iteratively repeated until no further compression of the signal occurred. The compression entropy, *E*_*c*_, was then obtained from:

(5) $$\begin{array}{l} E_{c}=Nlog_{2}\;M∕10, \end{array}$$

where *N* = number of symbols in the compressed message, *M* = number of different symbols in the message (Jiménez-Montano et al., 2000; French et al., 2003), division by 10 normalizes to the resolution of 10 bits. Theoretical maximum entropy of an action potential signal, *E*_max_, was estimated from:

(6) $$\begin{array}{l} E_{\text{max}}\approx F\;log_{2}\;\left(e.\Delta t∕F\right), \end{array}$$

where *F* is action potential firing rate, e is the exponential constant, and Δ*t* is the sample interval used (MacKay and McCulloch, 1952; Rieke et al., 1997). Theoretical entropy of regularly sampled Gaussian distributed random noise, *E*_G_, was estimated from:

(7) $$\begin{array}{l} E_{\text{G}}=log_{2}\left[{\left(2\pi \sigma^{2}e\right)}^{2}∕\Delta t\right], \end{array}$$

where σ^2^is the noise variance (French and Pfeiffer, 2011). *E*_G_ was estimated by assuming a maximum noise signal range of ±3σ.

### Statistical inference

Repeated measures of parameters fitted to frequency response functions, entropy, and information capacity were not normally distributed, as indicated by estimates of their skew and kurtosis. Comparisons of mean values of parameters under different conditions were made by the Wilcoxon signed-rank test for paired data and by the Mann–Whitney test for unpaired data (VassarStats: http://vassarstats.net/). All measurements are shown in the figures and tables as mean values and standard deviations. All data processing was performed by custom-written software written in the C++ language.

## Results

### Frequency responses during naturalistic and gaussian stimulation

Eleven different preparations were stimulated by both the naturalistic and Gaussian sequences. In each case the complete action potential records were obtained for both stimuli before applying TTX and proceeding with measurements of receptor potential and then receptor current to the two types of stimulation (Figure 1). Some preparations were lost before all experiments were completed, reducing the numbers of measurements of receptor potential and receptor current.

**Figure 2:**
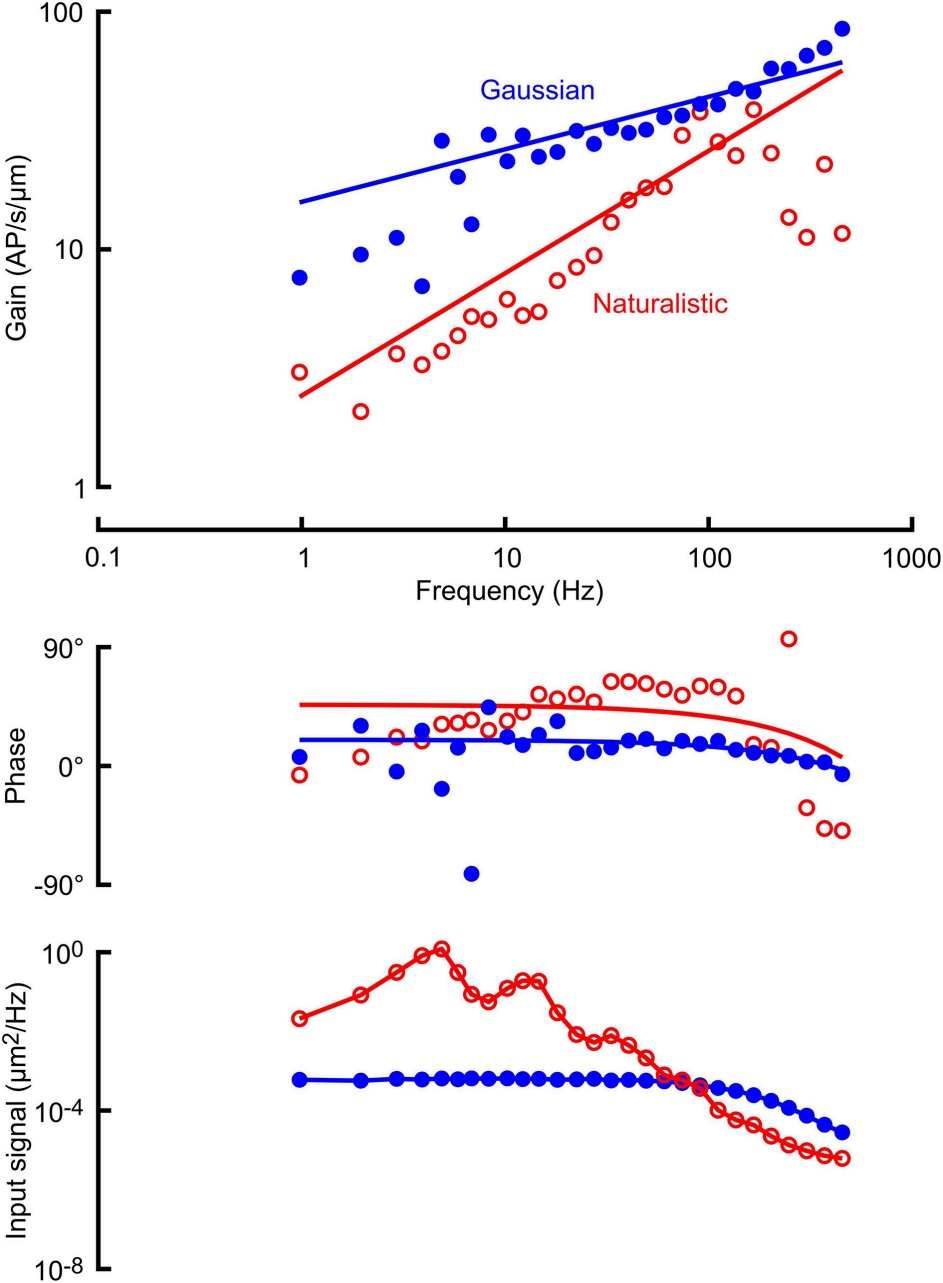
**Frequency response functions (gain and phase) between mechanical stimulation and action potentials in a VS-3 neuron using Gaussian noise (filled circles) and a naturalistic sequence (open circles)**. Solid lines through the gain and phase plots are the best fitting power law relationships (Equations 1 and 2) with parameters: *A* = 2.49 AP/μm.s, *k* = 0.50 (naturalistic) and *A* = 15.85 AP/μm.s, *k* = 0.22 (Gaussian). Lowest traces show the input signal powers, derived from the displacement sensor of the mechanical stimulator. The Gaussian signal was relatively flat until the response of the stimulator started to take effect above 70 Hz, and the naturalistic signal was strongly biased to lower frequencies, with several distinct peaks. Solid lines are drawn through the data points. Mean firing rates during these two experiments were 4.57 AP/s (naturalistic) and 5.92 AP/s (Gaussian).

Frequency response functions between mechanical stimulation and action potentials were well-fitted by the power-law relationship of Equations (1) and (2) (Figure 2; Table 1). Stimulus amplitudes were adjusted to make the mean action potential firing rates as close as possible during the naturalistic and Gaussian stimulation. Signal power of the naturalistic stimulation was concentrated in the frequency range below 100 Hz (bottom trace in Figure 2), whereas VS-3 neurons are increasingly sensitive to higher frequencies, so it was difficult to achieve mean firing rates above about 6 AP/s with naturalistic stimulation. Gaussian stimulation amplitude was adjusted to match this rate, and there was no significant difference between the mean firing rates for the two types of stimulation (Table 1). This preponderance of lower frequency stimulation also affected the quality of the naturalistic frequency response measurements, with greater variability at higher frequencies (Figure 2).

**Table 1:** **Fitted parameters to frequency response functions of naturalistic and Gaussian noise stimulated neurons (all values mean and standard deviation)**.

**Signal**	**Parameter**	**Naturalistic**	**Gaussian noise**	***p***
Receptor current	α (pA/μm)	3.84 (SD2.12, *n* = 9)	9.41 (SD6.20, *n* = 8)	0.0488
	*x*/λ		2.27 (SD0.54)	
Receptor potential	α (mV/μm)	1.29 (SD0.53, *n* = 10)	3.50 (SD2.58, *n* = 8)	0.0067
	*x*/λ		1.50 (SD0.36)	
Action potentials	*A* (AP/μm.s)	2.56 (SD1.21, *n* = 11)	20.01 (SD15.16, *n* = 11)	0.0036
	*K*	0.56 (SD0.08)	0.27 (SD0.09)	0.0036
	Rate (AP/s)	5.46 (SD2.63)	6.06 (SD4.09)	0.4654

**Figure 3:**
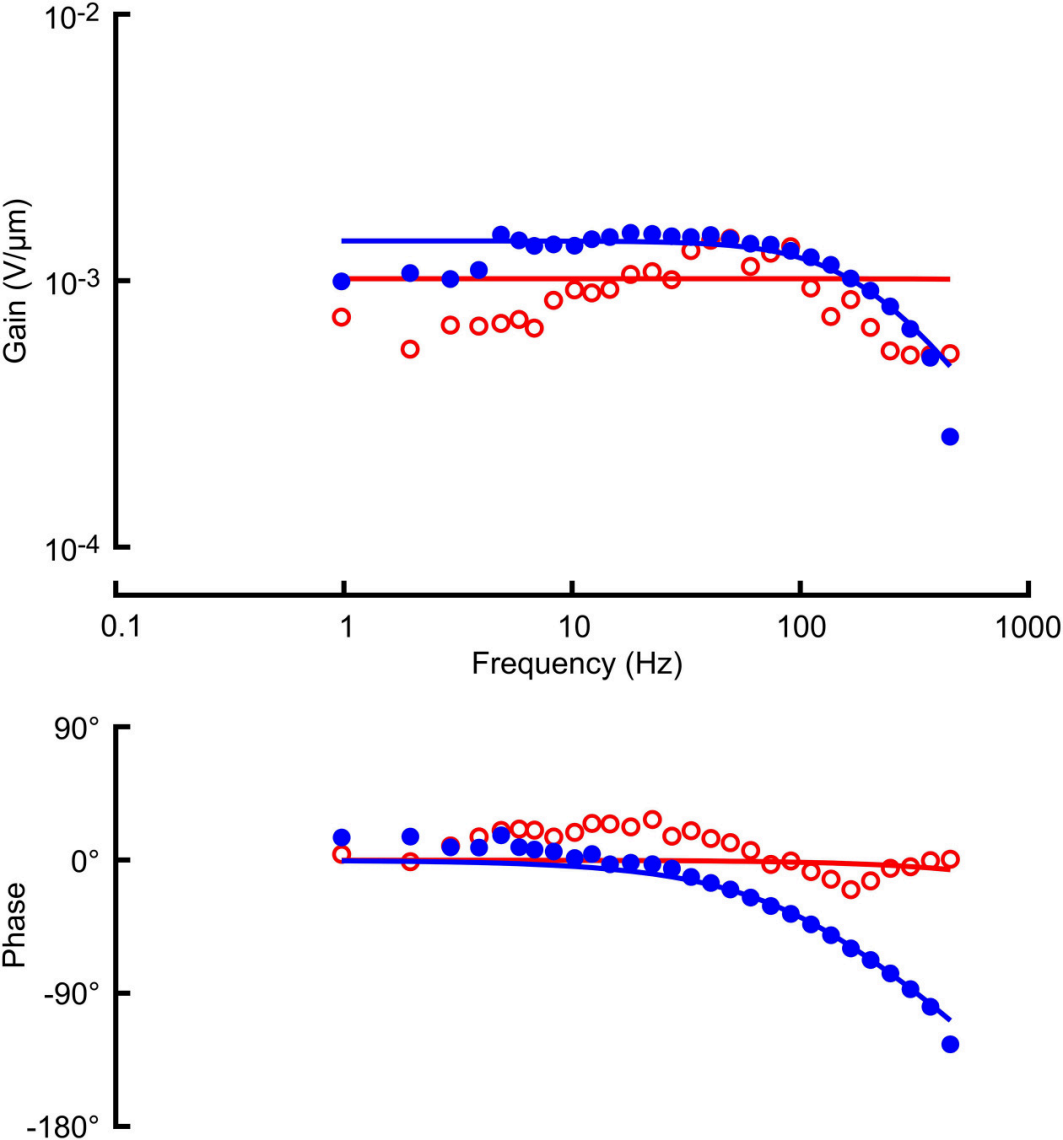
**Frequency response functions (gain and phase) between mechanical stimulation and receptor potential in the same VS-3 neuron as Figure 2, using Gaussian noise (filled circles) and a naturalistic sequence (open circles)**. Solid lines through the gain and phase plots are the best fitting cable relationships (Equation 3) with parameters: α = 1.02 mV/μm (naturalistic) and α = 1.71 mV/μm, l/λ = 1.54 (Gaussian). Naturalistic data could not be fitted well enough to give estimates of cable length constant, so only the mean sensitivity was recorded.

**Figure 4:**
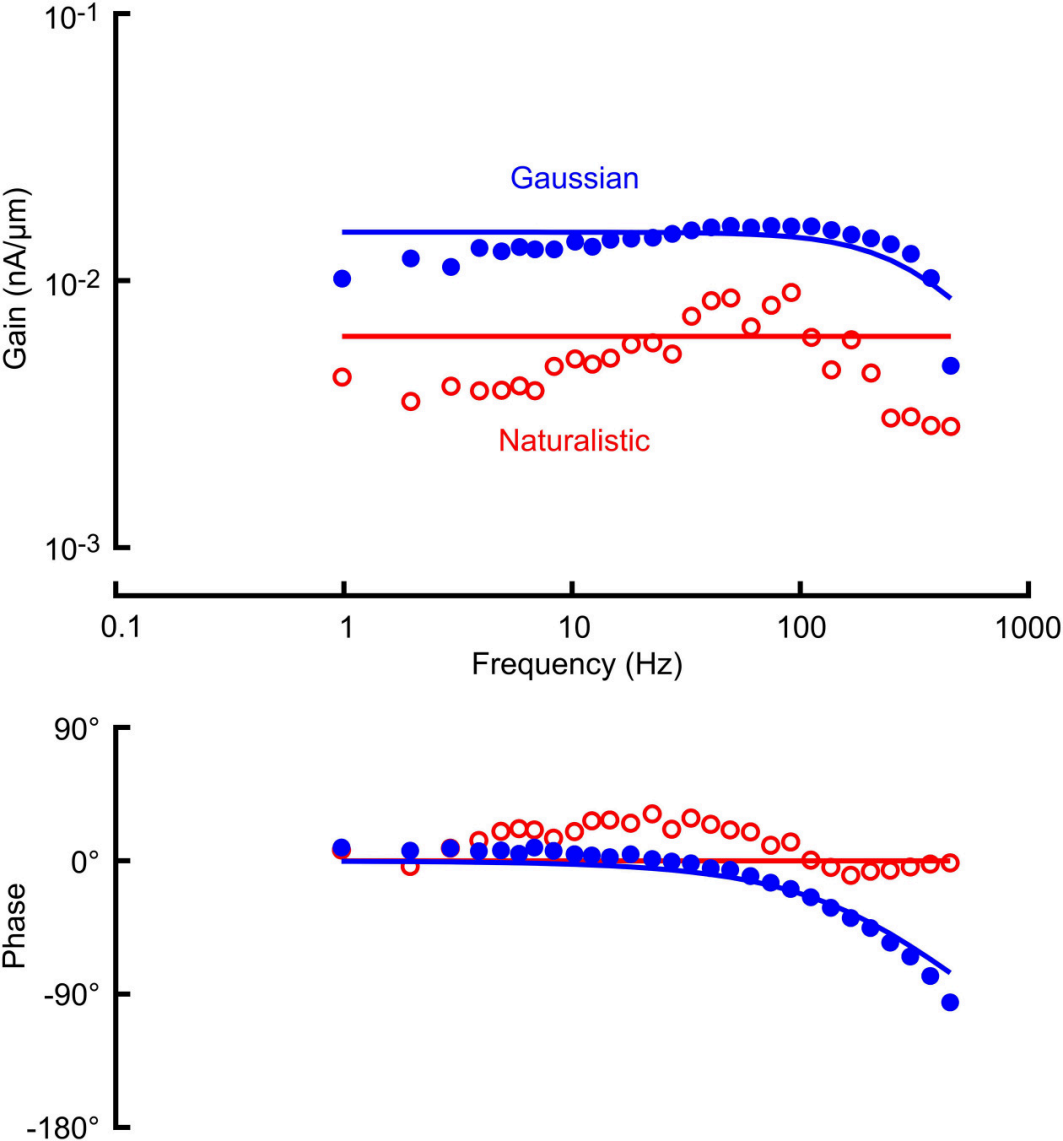
**Frequency response functions (gain and phase) between mechanical stimulation and receptor current in a VS-3 neuron using Gaussian noise (filled circles) and a naturalistic sequence (open circles)**. Solid lines through the gain and phase plots are the best fitting cable relationships (Equation 3) with parameters: α = 6.22 pA/μm (naturalistic) and α = 17.0 pA/μm, l/λ = 2.16 (Gaussian). As in Figure 3 only mean sensitivity could be recorded from naturalistic data.

Receptor potential and receptor current recordings were made with the same amplitudes of stimulation used for the action potentials (Figures 3, 4; Table 1). Both measurements were fitted well by the cable model (Equation 4) although the wider frequency range of the Gaussian stimulation again gave more reliable data. Receptor current data were more variable than receptor potential, reflecting the difficulty of voltage clamping the dendritic cable. It was difficult to fit Equation 4 reliably to the naturalistic receptor potential (Figure 3) and impossible for receptor current (Figure 4), so we recorded only the mean sensitivity over the complete frequency range (Table 1).

**Figure 5:**
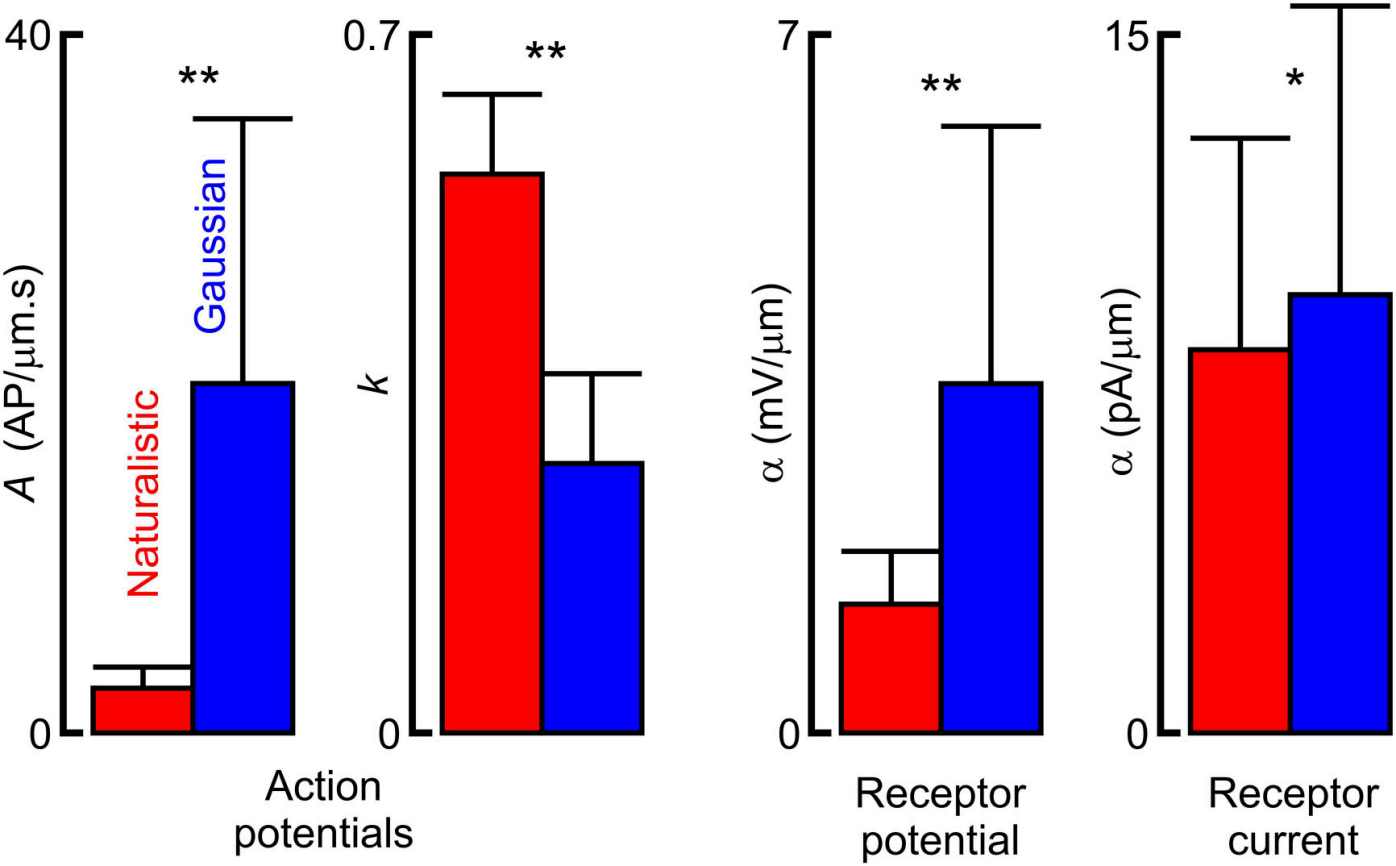
**Comparison of fitted parameters to frequency response functions from naturalistic vs. Gaussian noise stimulation**. Asterisks indicate statistical significance values of Wilcoxon (action potentials) and Mann–Whitney (receptor current and potential) nonparametric tests for difference between distributions of dependent and independent samples respectively (^*^p < 0.05, ^**^p < 0.01). Actual parameter values are given in Table 1.

Fitted frequency response parameters were significantly different during naturalistic vs. Gaussian stimulation (Figure 5; Table 1). Naturalistic stimulation gave higher values of the power-law exponent, *k*, representing a shift to higher frequency sensitivity, or faster adaptation. Since firing rates were the same, this change was accompanied by a reduction in low frequency sensitivity, shown by parameter *A*, the gain at 1 Hz. Receptor potential measurements indicated lower sensitivity but higher values of *x*/λ during naturalistic stimulation, but these data must be treated cautiously because of the difficulty of fitting naturalistic responses. The dendritic lengths, *x*, were clearly constant under the two conditions, so any change in *x*/λ would imply a change in the ratio of axial to radial cable resistance.

### Information measures during naturalistic and gaussian stimulation

**Figure 6:**
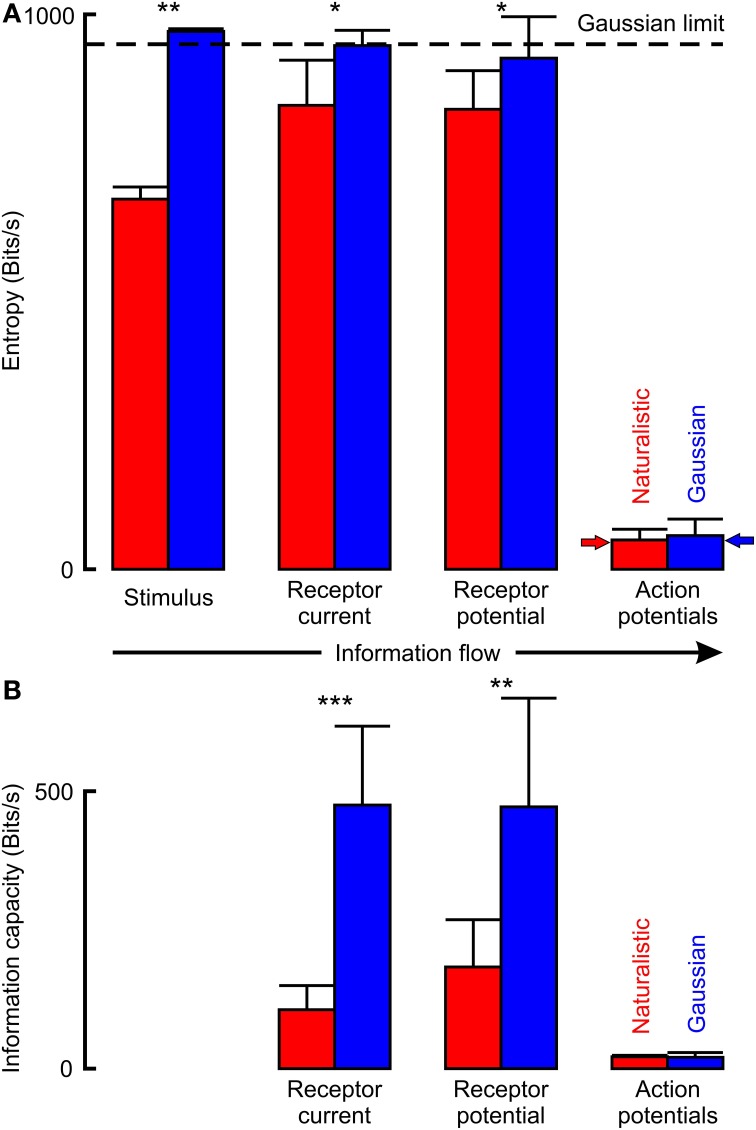
**Information flow through VS-3 neurons estimated by entropy (A) and by linear information capacity (B)**. Naturalistic stimulation used only part of the available entropy space that is theoretically available for Gaussian noise, shown as a dashed horizontal line. Entropy values for action potentials were much lower than the other signals, but close to the predicted maximum entropy rates (solid arrows). Actual parameter values are given in Table 2 (^*^p < 0.05, ^**^p < 0.01, ^***^p < 0.001).

The naturalistic stimulus had lower total entropy than the Gaussian stimulus that gave the same firing rate (Figure 6; Table 2). Gaussian stimulation produced receptor potential and receptor current signals that were all similar, and close to the theoretical limit for the entropy of a Gaussian distributed signal. Total entropy increased from naturalistic stimulation to receptor current and was then unchanged in receptor potential, indicating that uncorrelated noise was added during transduction. However, entropy during naturalistic stimulation was significantly lower than for Gaussian stimulation at each stage.

**Table 2:** **Entropy and information capacity of naturalistic and Gaussian noise stimulated neurons at each stage of mechanosensation (all values in Bits/s, mean and standard deviation)**.

**Signal**	**Measure**	**Naturalistic**	**Gaussian noise**	***p***
Stimulus	Entropy	667.3 (SD21.9, *n* = 11)	969.3 (SD4.8, *n* = 11)	0.0036
Receptor current	Entropy	836.6 (SD81.4, *n* = 9)	943.7 (SD27.6 *n* = 8)	0.0108
	Info.Cap.	106.2 (SD43.5)	475.0 (SD142.2)	0.0006
Receptor potential	Entropy	828.9 (SD69.7, *n* = 10)	921.1 (SD74.7, *n* = 8)	0.0232
	Info.Cap.	183.2 (SD 85.2)	471.7 (SD196.0)	0.0021
Action potentials	Entropy	52.86 (SD19.21, *n* = 11)	60.06 (SD 29.77, *n* = 11)	0.1615
	Info.Cap.	21.14 (SD2.52)	20.34 (SD8.63)	0.6101

Entropies of action potential signals were much lower than the preceding analog signals and not significantly different between naturalistic and Gaussian stimuli. They were also close to the predicted maximum entropies for binary signals at the given firing rates (small arrows in Figure 6), indicating that entropies were entirely limited by the firing rates.

Linear information capacities for Gaussian stimulation were approximately half the entropy values for the same signals (Figure 6; Table 2). Information capacities during naturalistic stimulation were significantly lower than for Gaussian, again indicating the addition of uncorrelated noise. Information capacities for action potential signals were less than half the equivalent entropy values, and again there was no significant difference between the two types of stimulation.

### Adapted vs. unadapted neurons

**Figure 7:**
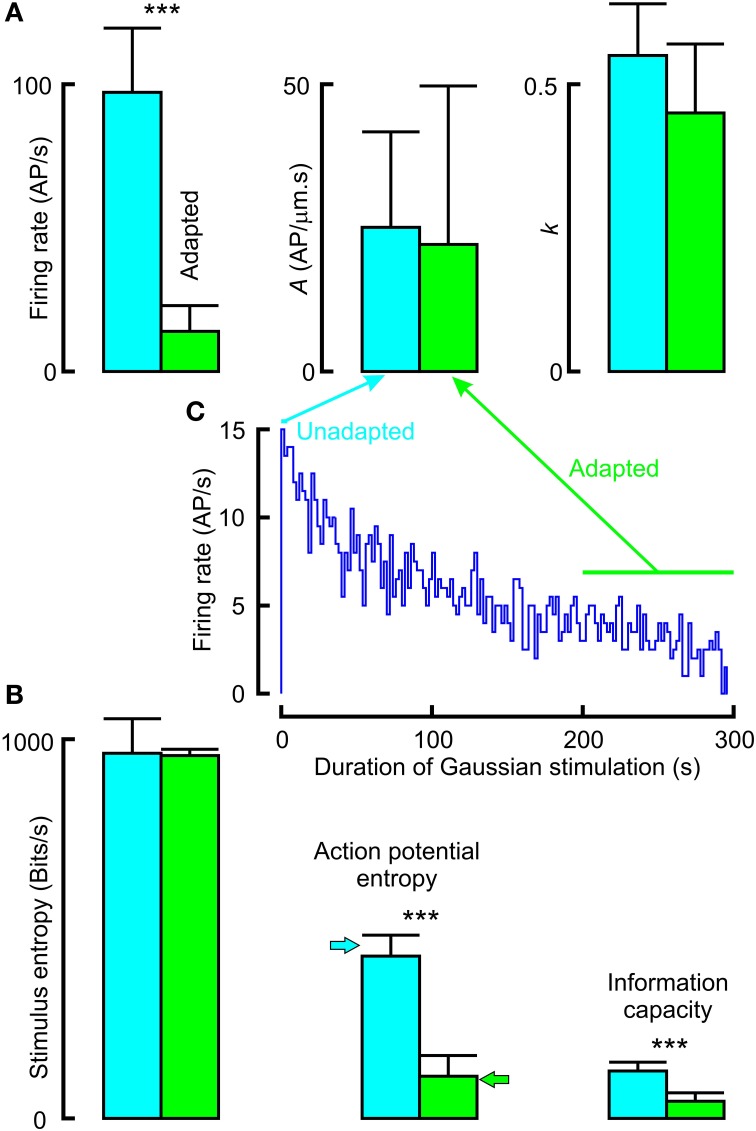
**Effects of adaptation to Gaussian noise on frequency response (A) and information transmission (B)**. Firing rate declined over a period of several minutes after commencing stimulation (inset histogram **C**). Unadapted data were collected during repeated presentation of noise for 4 s, with interspersed recovery periods. Adapted data were collected during the period of 200–300 s stimulation. Firing rate, entropy and information capacity were all significantly higher during unadapted vs. adapted periods, but frequency response parameters were not significantly different. Actual parameters in Table 3 (^***^p < 0.001).

Application of a randomly distributed mechanical stimulus of adequate amplitude produces continuous action potential firing in VS-3 neurons, but the rate of firing declines over a period of several minutes (Pfeiffer et al., 2009). We made recordings in which neurons were stimulated with Gaussian noise for only 4 s, and then allowed to recover for 25 s before the stimulation was repeated. Data from these, relatively unadapted, neurons were compared to data from adapted neurons after continuous stimulation for 200 s (Figure 7; Table 3). Mean firing rates for unadapted neurons were almost seven times higher than for adapted neurons, yet there were no significant differences between the fitted power-law parameters.

**Table 3:** **Frequency response, entropy and information capacity of action potentials in unadapted and adapted Gaussian noise stimulated neurons (all values mean and standard deviation)**.

**Measure**	**Unadapted**	**Adapted**	***p***
Firing rate (AP/s)	97.18 (SD22.35, *n* = 11)	13.97 (SD 8.97, *n* = 8)	0.0003
*A* (AP/μm.s)	25.09 (SD16.62)	22.11 (SD27.59)	0.1074
*K*	0.55 (SD0.09)	0.45 (SD0.12)	0.1471
Stimulus entropy (Bits/s)	962.88 (SD9.11)	956.82 (SD16.66)	0.9681
Action potential entropy (Bits/s)	428.78 (SD54.44)	111.13 (SD54.71)	0.0003
Information capacity (Bits/s)	125.15 (SD23.28)	45.19 (SD22.23)	0.0003

Stimulus entropy for the two conditions was the same, and close to the Gaussian limit, as expected. However, action potential entropy was significantly lower in the adapted neurons, and close to the maximum entropy values predicted for the firing rates (Figure 7). Linear information capacities were again lower than the entropy values, and significantly lower in the adapted, vs. unadapted, neurons.

## Discussion

### Naturalistic signals change VS-3 neuron dynamic sensitivity

Real or simulated naturalistic input signals have been used to examine sensory dynamics in a relatively small number of previous studies (Rieke et al., 1995; van Hateren, 1997; Juusola and de Polavieja, 2003; Dupuy et al., 2012). We found that naturalistic signals produced by locust walking were characterized by relatively low frequencies (< 100 Hz) and by several prominent frequency peaks (Figure 2). These properties are not surprising from the rhythmic walking of an insect, and other naturalistic signals have also shown restricted frequency ranges, prominent peaks in frequency, and wide dynamic range (Rieke et al., 1995; van Hateren, 1997) that may be expected to affect measurements from nonlinear dynamic systems. The major effect that we observed was an increase in high frequency sensitivity in the action potential signals, as measured by the power-law exponent, *k* (Figures 2, 5). This was accompanied by a significant reduction in the sensitivity at 1 Hz, *A*, which may be explained by our requirement that the naturalistic and Gaussian signals produce similar firing rates. Since most action potential firing in these rapidly adapting neurons is produced by higher input frequencies, increasing *k* while reducing *A* results in similar signal power levels at these higher frequencies, as seen in Figure 2.

Increasing high frequency sensitivity when the input signal contains less higher frequencies suggests a general mechanism for extracting maximum information about less well represented frequencies in the input signal. This could be functionally useful; allowing neurons to dynamically adjust their sensitivity within frequency bands to retain adequate sensitivities over a wide range of frequencies. The dynamic properties of action potential firing in VS-3 neurons are probably dominated by relatively slow inactivation and recovery from inactivation of voltage-activated sodium channels (Torkkeli and French, 2002), suggesting that the slower patterns of naturalistic, vs. Gaussian, signals interact with these time-dependent properties of sodium channel inactivation.

Significant sensitivity reductions were also seen in the receptor current and potential (Figures 3, 4, 5). Although these analog fluctuations would be expected to be much closer to linearity than action potentials, fluctuating receptor potentials could clearly modulate other ionic mechanisms that create nonlinear reductions in sensitivity, such as increasing membrane conductance to reduce the effect of receptor current. Analogous interactions between voltage-activated currents and receptor current via membrane potential were reported in fly photoreceptors (Niven et al., 2004). Receptor current should be less affected if the membrane potential is truly clamped, and mean receptor current was less reduced than receptor potential (Figure 5), but the difficulty of achieving complete voltage clamp at the end of a long sensory dendrite means that these data must be treated with caution. Values of *x*/λ for the receptor potentials were about 50% higher than the mean value reported previously (Gingl and French, 2003), but this parameter depends on dendritic length, which varies strongly across the slit organ. Fitting with a cable equation alone implies that the mechanotransduction process itself has significantly faster dynamics than the dendritic cable. This was supported by earlier voltage-jump experiments (Gingl and French, 2003) but has not yet been justified by more direct measurements.

### Action potential encoding limits final entropy and information capacity

During Gaussian noise stimulation the entropy in the input signal, receptor current, and receptor potential were close to the theoretical limit for a Gaussian signal (Equation 7; Figure 6), as found previously (French and Pfeiffer, 2011; Pfeiffer et al., 2012). However, action potential entropy was much lower, and limited by the maximum entropy for binary signals (Equation 6; Rieke et al., 1997; Pfeiffer and French, 2009). Entropy in the naturalistic signal was significantly below the theoretical limit. Increased levels were seen in the receptor current and receptor potential, although still significantly less than with Gaussian stimulation. This extra entropy probably represents addition of uncorrelated noise to the receptor current. Such uncorrelated noise must also have been added to the Gaussian stimulated neurons, but could not increase total entropy because of the theoretical limit, but instead effectively replaced original input signal. Entropy in the naturalistically stimulated action potentials was much lower, and not significantly different from the Gaussian stimulation, again close to the theoretical maximum entropy for a binary signal at the firing rate.

Linear information capacity of Gaussian stimulated neurons was about half the entropy values. For receptor current and potential these values should be dominated by signal-to-noise ratios, supporting the idea that significant uncorrelated noise is added during initial transduction of displacement to current. Information capacities of naturalistically stimulated neurons were even lower, suggesting that a significant amount of the added noise is in the lower frequency range of the stimulus. It has consistently been found that linear information capacity of graded neural signals are about one order of magnitude higher than action potential signals in the same, or closely related neurons (Juusola and French, 1997; DiCaprio et al., 2007). Action potential information capacities here were similar to values reported at these firing rates previously (French et al., 2001) and did not significantly depend on stimulation, again supporting the view that firing rate is the limiting factor.

### Adaptation reduces information transmission

The naturalistic stimulus that we used was clearly different to Gaussian random noise in its frequency components, but naturalistic signals could differ from random in other properties, including the levels of adaptation and firing rate that they cause in a sensory neuron. The adaptation experiments (Figure 7) showed that this was not the case here because dynamic properties of the action potential signal did not depend on firing rate or other effects of adaptation. Both the power law exponent, *k*, and the overall sensitivity, *A*, were independent of adaptation to the lower firing rate. However, firing rate does affect information transmission; both entropy and information capacity were significantly lower when the firing had slowed after 200 s, even though the neurons were receiving the same Gaussian noise signal. Entropy values were always close to the theoretical values for a binary signal.

### Relationship to living vibratory communication

Barth (2002) reviewed the possible roles of *Cupiennius* slit-sense organs in detail, and from several perspectives, including body locations, mechanical linkage to the substrate, and the behavioral responses. He also reviewed the types of natural vibration that reach the animals from prey species and mating partners, including those via plant substrates. Spiking responses of neurons in a metatarsal slit-sense organ to mating vibrations have also been observed (Molina et al., 2009). These previous studies also show that natural stimuli do not resemble random noise, but instead tend to have prominent peaks at one or more dominant frequencies, which was also the case for the stimuli we used here.

The main purpose of the present study was to test the hypothesis that nonlinearities in mechanotransduction change the dynamic response of VS-3 neurons. This was clearly shown by the linear frequency response measurements (Figure 2), and deserves further examination by nonlinear systems analysis to seek the nature and origin of the nonlinearity. The increase in sensitivity to higher frequencies that we found with the naturalistic input would probably favor the detection of pulsed displacements, such as those recorded during mating (Barth, 2002; Molina et al., 2009).

### Conclusions

Naturalistic stimulation affected signal transmission at each stage of mechanosensation (conversion of displacement to action potentials), but the strongest effect was a change in the dynamic properties of the action potential encoder, with lower frequency signals causing higher frequency sensitivity. Uncorrelated noise is added during transduction, but total information in the action potentials is primarily limited by the maximum entropy that can be encoded in these binary signals.

### Conflict of interest statement

The authors declare that the research was conducted in the absence of any commercial or financial relationships that could be construed as a potential conflict of interest.
